# Application of a Convolutional Neural Network for Multitask Learning to Simultaneously Predict Microvascular Invasion and Vessels that Encapsulate Tumor Clusters in Hepatocellular Carcinoma

**DOI:** 10.1245/s10434-022-12000-6

**Published:** 2022-06-26

**Authors:** Tongjia Chu, Chen Zhao, Jian Zhang, Kehang Duan, Mingyang Li, Tianqi Zhang, Shengnan Lv, Huan Liu, Feng Wei

**Affiliations:** 1 Department of Hepatobiliary and Pancreatic Surgery, The First Hospital of Jilin University, Changchun, People’s Republic of China; 2 College of Computer Science and Technology, Jilin University, Changchun, People’s Republic of China; 3 Department of Radiology, The First Hospital of Jilin University, Changchun, People’s Republic of China

## Abstract

**Background:**

Hepatocellular carcinoma (HCC) is the fourth most common cause of cancer death worldwide, and the prognosis remains dismal. In this study, two pivotal factors, microvascular invasion (MVI) and vessels encapsulating tumor clusters (VETC) were preoperatively predicted simultaneously to assess prognosis.

**Methods:**

A total of 133 HCC patients who underwent surgical resection and preoperative gadolinium ethoxybenzyl-diethylenetriaminepentaacetic acid (Gd-EOB-DTPA)-enhanced magnetic resonance imaging (MRI) were included. The statuses of MVI and VETC were obtained from the pathological report and CD34 immunohistochemistry, respectively. A three-dimensional convolutional neural network (3D CNN) for single-task learning aimed at MVI prediction and for multitask learning aimed at simultaneous prediction of MVI and VETC was established by using multiphase Gd-EOB-DTPA-enhanced MRI.

**Results:**

The 3D CNN for single-task learning achieved an area under receiver operating characteristics curve (AUC) of 0.896 (95% CI: 0.797–0.994). Multitask learning with simultaneous extraction of MVI and VETC features improved the performance of MVI prediction, with an AUC value of 0.917 (95% CI: 0.825–1.000), and achieved an AUC value of 0.860 (95% CI: 0.728–0.993) for the VETC prediction. The multitask learning framework could stratify high- and low-risk groups regarding overall survival (*p* < 0.0001) and recurrence-free survival (*p* < 0.0001), revealing that patients with MVI+/VETC+ were associated with poor prognosis.

**Conclusions:**

A deep learning framework based on 3D CNN for multitask learning to predict MVI and VETC simultaneously could improve the performance of MVI prediction while assessing the VETC status. This combined prediction can stratify prognosis and enable individualized prognostication in HCC patients before curative resection.

**Supplementary Information:**

The online version contains supplementary material available at 10.1245/s10434-022-12000-6.

Hepatocellular carcinoma (HCC) accounts for more than 80% of the primary liver cancers and is the fourth most common cause of cancer-related death worldwide.^1,2^ However, due to the high risk of early relapse and metastasis, the prognosis is dismal. Therefore, predicting the prognosis is necessary for the formulation of a treatment plan.

Microvascular invasion (MVI), as an important part of the hypothesis, is a well-known prognostic factor occurring after surgical resection in patients with HCC.^3,4^ The incidence of MVI can reach 15–57.1%,^5^ and recent studies^3,6,7^ have shown that MVI is the main risk factor for early recurrence in the first 2 years after curative treatment. Therefore, MVI could be used as a predictor of early recurrence in patients with HCC after hepatectomy. The determination of MVI is traditionally based on the microscopic examination of postoperative specimens, and surgeons are usually unable to assess this before surgery. Previously, some studies^8–12^ have begun to use preoperative data as input to establish models to predict MVI before hepatectomy. Preoperative prediction of MVI is proposed to be critical when developing treatment strategies for improved therapeutic outcomes in patients with HCC. However, many patients without MVI experience early recurrence after hepatectomy, which challenges the heterogeneity and mechanism of MVI in predicting HCC recurrence and metastasis.

Vessels encapsulating tumor clusters (VETC), affecting the recurrence-free survival (RFS) and overall survival (OS) of HCC patients,^13^ is present in some patients without MVI, and these patients have a worse prognosis than those without either MVI or VETC.^14^ Noting that VETC has complementary significance to MVI in predicting prognosis to some extent, recent studies began to use preoperative images to assess the VETC status.^15,16^ Additionally, a significant correlation between VETC and MVI has been presented.^13^ Lin et al.^17^ identified VETC and MVI as independent predictors for RFS and incorporated them into a multivariate model to predict RFS. Lu et al.^14^ determined the prognostic role of a novel vascular classification system based on the VETC and MVI statuses after curative resection. A certain degree of relevance exists between MVI and VETC, which could be combined as a preoperative prediction label for survival. However, the specific effect of the label combination in predicting prognosis remains unclear.

Developments in imaging technology and artificial intelligence have enabled preoperative noninvasive assessments of MVI.^18,19^ Deep learning (DL) technology can combine the low-level feature representation of the task into higher-level semantic features through the deep structure of multiple hidden layers to achieve the learning goal of the model. It performs better than radiomics and has been proven to solve various challenging clinical problems.^20,21^ DL is increasingly applied to predict the MVI status but ignores the correlation between VETC and MVI.^22–25^ Thus, a multitask learning method was adopted to simultaneously predict MVI and VETC statuses. We introduced features that are helpful for predicting MVI in the process of predicting VETC through model-sharing parameters, and used the correlation between VETC and MVI to improve the performance of MVI prediction. By sharing the feature representation between two related tasks, the generalization ability of the multitask model in predicting MVI tasks is better than that of the single-task model.^26^

In this study, a three-dimensional convolutional neural network (3D CNN), as a suitable method for processing image data in the DL algorithm, was used for multitask learning of preoperative gadolinium ethoxybenzyl-diethylenetriaminepentaacetic acid (Gd-EOB-DTPA)-enhanced MRI data of HCC patients to predict MVI and VETC statuses simultaneously. The 3D CNNs could potentially assist surgeons in formulating treatment strategies and accurately assess prognosis.

## Methods

### Study Population

**Fig. 1:**
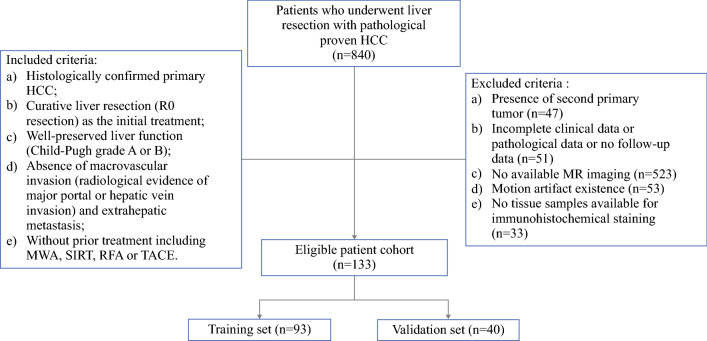
Flow chart of patient recruitment for the study. *HCC*, hepatocellular carcinoma

The present study was approved by the institutional ethical review board of the First Bethune Hospital of Jilin University. It was conducted in accordance with the ethical guidelines of the 1975 Declaration of Helsinki.^27^ From January 2017 to August 2020, a total of 840 consecutive patients underwent partial hepatectomy and were diagnosed with HCC based on pathological results in our center. The relevant patient information was extracted from the case resource database. The patient recruitment pathway and the inclusion and exclusion criteria are listed in Fig. 1. Finally, 133 patients were included. The patients were randomly stratified into a training set (*N* = 93; 35 [37.6%] MVI-positive cases, 31 [33.3%] VETC-positive cases) and a validation set (*N* = 40; 16 [40.0%] MVI-positive cases, 13 [32.5%] VETC-positive cases) at a ratio of 7:3.

### MRI Protocol

The enhanced MR images included in this study were obtained by three different MRI scanners, including a Discovery MR750 3.0 T (GE Healthcare, USA) with breath-hold axial liver acquisition with volume acceleration (LAVA), an Ingenia 3.0 T (Philips Medical Systems, Netherlands), and an Achieva 3.0 T (Philips Medical Systems, Netherlands) with axial enhanced T1 high-resolution isotropic volume excitation sequence (e-THRIVE) protocols (Supplementary Table 1). The contrast agent used was Primovist (Bayer, Germany), the bolus injection rate was set to 1 ml/s, and the contrast dose for each patient was 25 μmol/kg body weight (0.1 ml/kg). Subsequently, 15–20 ml of saline was flushed at a rate of 2 ml/s for each patient. Enhanced MR images of the late arterial phase (L-AP), portal vein phase (PVP) and hepatobiliary phase (HBP) were obtained 30 s, 45 s, and 20 min after the injection of the contrast agent.

### Volumetric Region Extraction and Data Augmentation

**Fig. 2:**
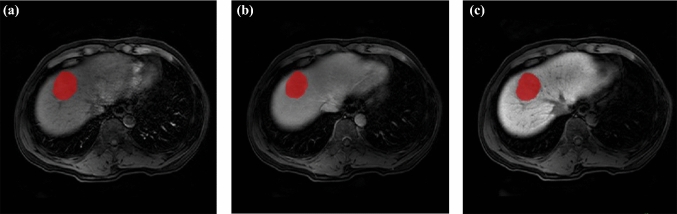
A case of hepatocellular carcinoma (HCC) with Gd-EOB-DTPA-enhanced MRI: a 50-year-old man with pathologically confirmed HCC. The lesion area on each axial slice of arterial phase **a**, portal vein phase **b**, and hepatobiliary phase **c** images was delineated

The regions of interest (ROIs) of HCC (Fig. 2) were determined by two radiologists separately using ITK-SNAP software (http://www.radiantviewer.com). The ROI was manually delineated on each axial slice of the AP, PVP, and HBP images, covering the entire tumor. Finally, a volume of interest (VOI) representing the tumor area was manually extracted. Before inputting into the models, the segmentation results were independently validated by a senior radiologist to reduce possible bias. In the training stage, the data in the training set were augmented by random rotation, random horizontal flipping, random vertical flipping, affine transformation, and elastic transformation, and the data were expanded to twice the amount of the training set data. The data in the test set were not augmented.

### Confirmation of MVI and VETC Statuses

**Fig. 3:**
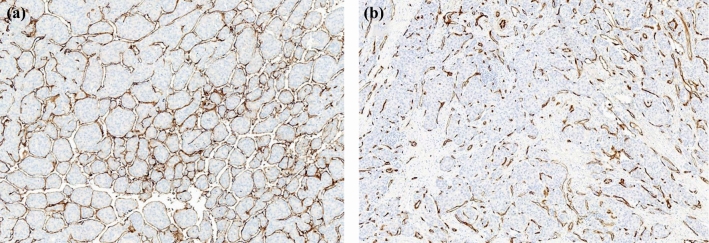
Representative morphological features of VETC status in HCC. **a** VETC-positive, **b** VETC-negative

The determination of the presence of MVI in HCC patients depends on the reading of MVI status described in the pathological report after hepatectomy. The status was reviewed by two senior pathologists who were both blinded to the clinical data. In the routine postoperative pathology report, there was no record of VETC status. Therefore, we obtained at least 3 tissue blocks from different parts of the tumor acquired from each selected patient, with a mean of 4.1 (median 4, range 3–5) paraffin-embedded tissue blocks per tumor available for evaluation. Immunohistochemistry following the manufacturer’s instructions was performed on all specimens. In brief, paraffin embedded slices were dewaxed, rehydrated and were subsequently tested for antigens. BSA (3%) was used for blocking in the dark for 30 min. The slices were incubated with CD34 antibody (CST, ICO115) overnight at 4 °C and incubated with a secondary antibody for 50 min afterward. After treated with DAB and hematoxylin to re-stain the nuclei, slide images were taken using a Nikon E100 microscope. Considering the definition of VETC, the area of VETC was semi-quantitatively evaluated in 5% of the units according to the CD34 evaluation, as the degree of the VETC-positive area ranged from 0% to 100% of the tumor region; 55% was considered the optimal cutoff value to further divide HCC patients into VETC+ and VETC− groups as previously reported^13^ (Fig. 3).

### Training a DL Model for Single-Task Learning (STL)

**Fig. 4:**
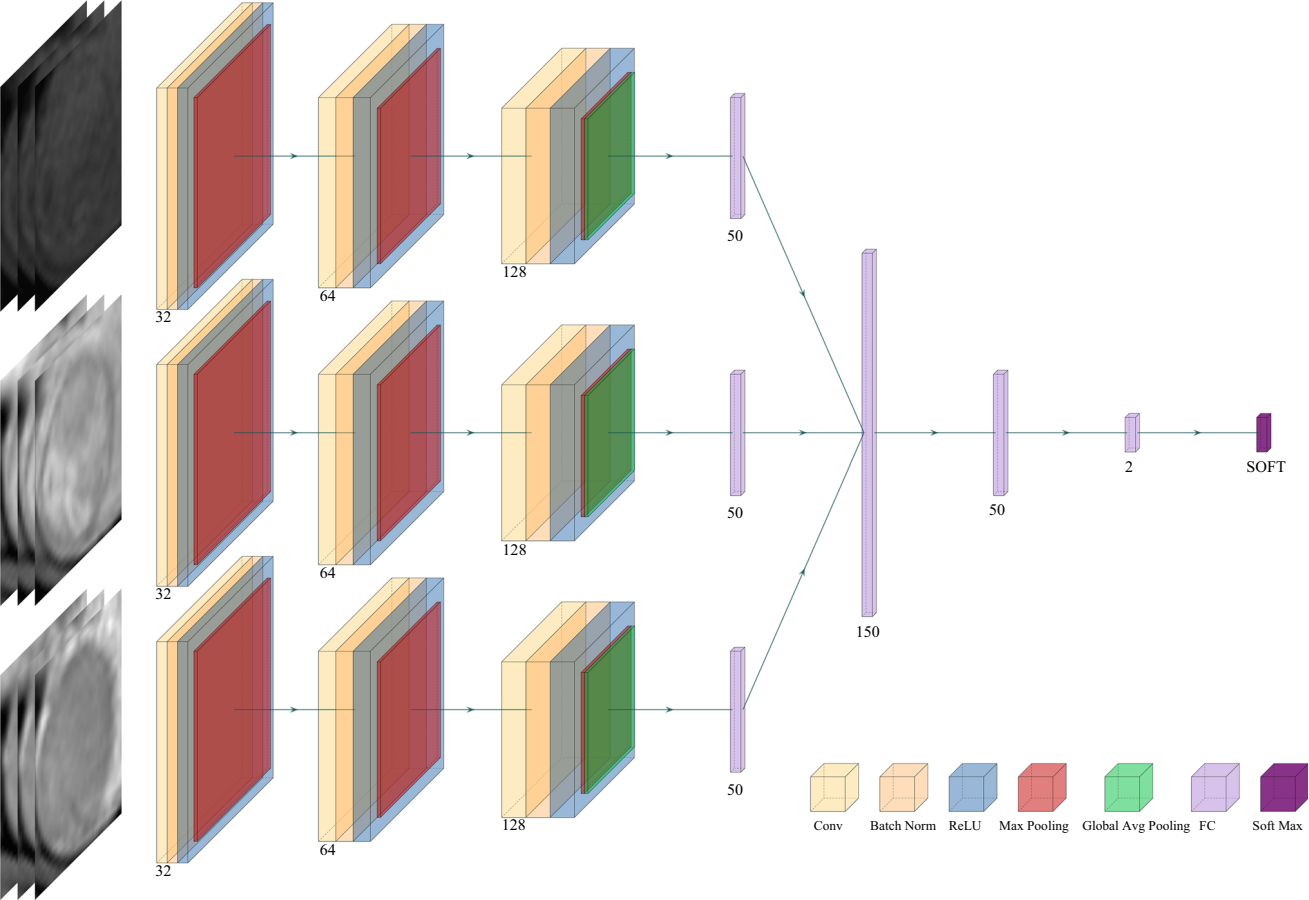
Flowchart of the proposed deep learning framework for single-task learning

Figure 4 shows the designed network structure of the DL model for STL, which uses 3D CNN to extract deep features related to MVI characterization from three MRI sequences. First, all voxels in the MRI were normalized, and the lesion area was then cut into a cube shape according to each VOI. Due to the different sizes of tumors, all extracted cube regions were normalized to a preset size of 60 × 60 × 60. The Adam algorithm was used as the optimization algorithm in the training phase, the batch size in training was set to 16, and the model learning rate was 10^-4^. For the structure of the model, three-phase MRI images were input at the same time. Each phase extracted features through a 3D CNN, and then the extracted three-phase feature vectors were concatenated and input into the fully connected layer. Finally, the softmax layer outputs the predicted MVI status. The 3D convolution operation (3 × 3 × 3, stride =1, padding=1) was applied to extract feature maps containing feature information. Each convolution operation was followed by the batch normal layer, the ReLU layer, and the max pooling layer. The batch normal layer aimed to speed up the convergence of the model and prevent the model from overfitting. The max pooling layer (2 × 2 × 2, stride = 2) was used to reduce the dimensionality of the data so that the model could extract data features from different levels of receptive fields. After the last convolution operation, global average pooling was used to convert the feature maps extracted by the CNN into feature vectors. Then, the feature vectors extracted in the three sequences were concatenated and input into the subsequent fully connected layer. A 2-dimensional one-hot vector was output through the softmax layer to indicate that the model finally predicted whether MVI status was positive or negative.

### Training a DL Model for Multitask Learning (MTL)

**Fig. 5:**
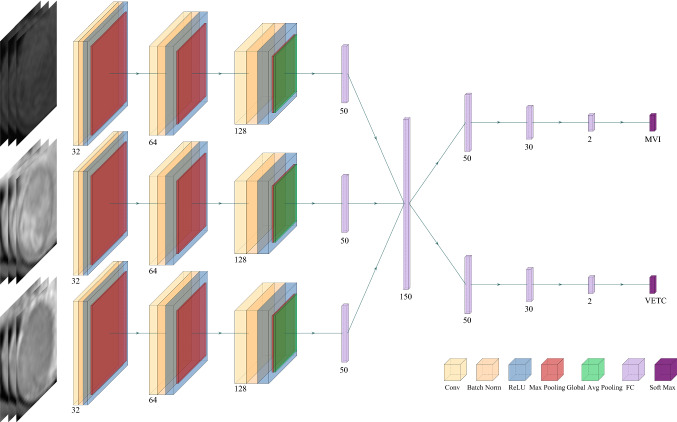
Flowchart of the proposed deep learning framework for multitask learning

Figure 5 shows the second model we trained, which used MTL to predict both MVI and VETC simultaneously. The model could be roughly divided into two steps. In the first step, the model input three phases of images at the same time, used the 3D CNN to extract the features, and then connected the feature vectors of the three phases into one feature vector. The two tasks of predicting MVI and VETC shared the same model parameters and shared the underlying features extracted by the model. In the second step, the model used its own unique model parameters according to the specific tasks to be performed. The first step enabled the model to make full use of the correlation between the two tasks, and the second step ensured the difference between the tasks. Thus, the accuracy of the model in predicting MVI was further improved, and the generalization ability of the model was enhanced.

### Statistical Analysis

We used the area under the receiver operating characteristic (ROC) curve (AUC), accuracy, specificity, sensitivity, precision, and F1-score for model evaluation. The chi-square test or Fisher’s exact test was used to analyze categorical variables, and the independent Student’s *t*-test or Mann–Whitney *U* test was used to analyze continuous data. Survival curves for each group were used to evaluate OS and RFS, as calculated by the Kaplan–Meier method, and were compared using the log-rank test. All statistical analyses were performed with PASW Statistics, version 18.0 (SPSS Inc., Chicago, IL, USA) and R software, version 3.4.1 [www.R-project.org (accessed on 30 June 2017)]. The threshold for statistical significance was a 2-sided *p* < 0.05.

## Results

### Demographic Comparison of Baseline Clinical Features

After a review of the postoperative pathology report and the immunohistochemistry results, among the 133 lesions, 51 were pathologically determined to have MVI present, while 44 were pathologically determined to have VETC present. To verify the performance of the 3D CNN for MTL, the dataset was divided into two parts, including 93 HCC patients in the training dataset and the remaining 40 HCC patients in the independent validation dataset. The clinical characteristics of the patients in the training and validation cohorts are summarized in Supplementary Table 2.

### Predictive Performance of the DL Model

**Fig. 6:**
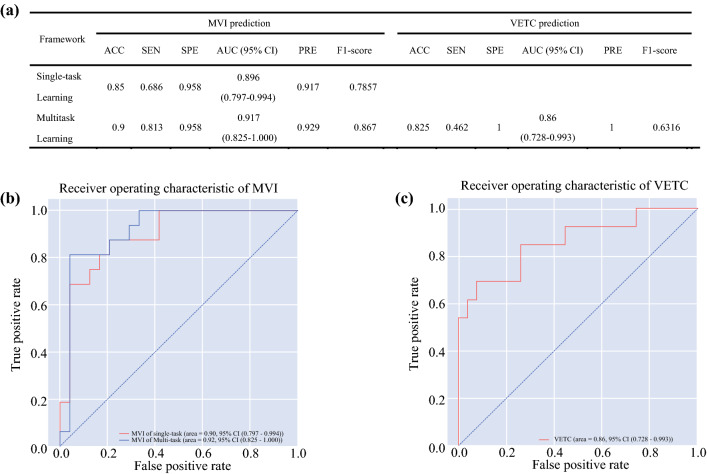
**a** Performance of 3D convolutional neural networks for single-task learning and multitask learning. **b** ROC curves of 3D convolutional neural networks (CNNs) for microvascular invasion (MVI) prediction in single-task learning and multitask learning. **c** ROC curves of 3D convolutional neural networks (CNNs) for vessels that encapsulate tumor cluster (VETC) prediction in multitask learning

The performance of the 3D CNN for predicting the corresponding target was separately assessed. As shown in Fig. 6a, the proposed method of STL yielded moderate performance, with an accuracy of 0.85 and an AUC of 0.896 [95% confidence interval (CI): 0.797–0.994]. The MTL model yielded better performance with an accuracy of 0.90 and a higher AUC of 0.917 (95% CI: 0.825–1.000). The sensitivity and specificity of the MTL model were also better than those of the STL model. The ROC curves based on 3D CNN for STL and MTL to predict MVI status are plotted in Fig. 6b. The 3D CNN of MTL produced good performance in predicting VETC status simultaneously, with an accuracy of 0.825 and an AUC of 0.8604 (95% CI: 0.728–0.993). The ROC curves based on 3D CNN for MTL to predict VETC status are plotted in Fig. 6c.

### Prognostic Assessment of the DL Model

**Fig. 7:**
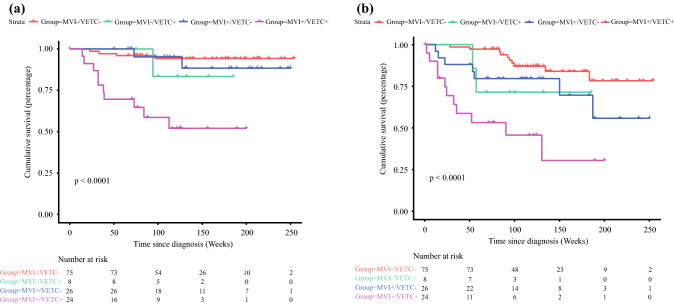
Overall survival (OS) and recurrence-free survival (RFS) curves scaled by MVI-VETC status predicted by the 3D CNN for MTL with Kaplan–Meier analysis. **a** Overall survival. **b** Disease-free survival

We further evaluated the prognostic role of the VETC-MVI model in prognosis. According to the output of the MTL model, patients could be stratified into VETC+/MVI+, VETC−/MVI−, VETC+/MVI−, and VETC−/MVI+ subpopulations. For OS and RFS, the MTL model showed meaningful performance (OS: *p* = 0.01, PFS: *p* < 0.001). The postoperative PFS and OS were significantly different between the MTL-predicted VETC+/MVI+ group and some other groups (VETC+/MVI+ vs VETC−/MVI−, OS: *p* < 0.001, PFS: *p* < 0.001; VETC+/MVI+ vs VETC+/MVI−, OS: *p* = 0.002, PFS: *p* = 0.026). The Kaplan–Meier curves are shown in Fig. 7.

## Discussion

HCC, as one of the most fatal diseases, has the highest incidence and mortality in East Asia and Africa.^28^ Patients with HCC are highly heterogeneous, and the outcomes after radical treatment are different. It is difficult for surgeons to assess recurrence after surgery. How to evaluate prognosis before surgery has remained an unsolved problem. As one of the most powerful preoperative diagnostic tools, imaging data of HCC plays a significant role in MVI and VETC prediction.

Contrast-enhanced MRI images were chosen to be included in the study according to the clinical practice guidelines of the National Comprehensive Cancer Network (NCCN).^29^ Theoretically, more information could be obtained, including anatomical and functional aspects, from MRI images. Therefore, Gd-EOB-DTPA-enhanced MRI images were selected as the input data. The choice of AP, PVP, and HBP MR sequences was based on their higher image quality and their diagnostic significance indicated in the clinical guidelines of the NCCN. The input of multisequence data would enable the model to grasp different information from each sequence, thereby improving the predictive performance.

The DL algorithm was applied as a tool instead of the conventional radiomics methods that require considerable manpower and time and rely on precise tumor contours delineated manually. The radiomics method is based on a hand-made feature extractor, which is usually affected by subjective factors to a certain extent.^12^ Feng et al.^10^ proposed a radiomics model using the HBP in Gd-EOB-DTPA-enhanced MRI to preoperatively predict MVI, which had an AUC value of 0.85. In the presence of subjective factors, the model does not achieve good results. However, the DL algorithm could use a cube area containing the tumor and peripheral tissue as the VOI without strict requirement on the boundary. When proceeding with data labeling, it was unnecessary to perform pixel-level tumor labeling, and cubes were used to label the general tumor position. This corresponds with the objective progression of malignant tumors and could reduce the subjective bias in the delineation process, especially in the application of small datasets for lesion characterization.^24^ Many studies have proven that MVI prediction could help surgeons assess the prognosis of patients before surgery and formulate more accurate treatment plans to improve prognosis.^22–25,30,31^ Each study used its own data for DL to predict MVI, which achieved AUCs ranging from 0.810 to 0.915. The prediction performance of the model was ensured by the change of the source data type and the DL algorithms.

MVI and VETC are two completely different vascular modes that both affect tumor metastasis. However, patients without MVI still experience recurrence and metastasis, and the use of MVI status in predicting prognosis is somewhat stretched. Therefore, we introduced VETC, which could explain the mechanism of Epithelial-mesenchymal transition (EMT) tumor cell metastasis^32^ and had a certain connection with MVI.^13,17,33^

Two types of models to predict MVI were trained in our research. The first model predicted MVI using a 3D CNN for STL with an input of multisequence MRI images, and produced a good performance, with an AUC of 0.896 (95% CI: 0.797–0.994). The second model was based on the idea of MTL using a 3D CNN to simultaneously predict MVI and VETC. Since the image data were taken from multiple models of equipment, we standardized the data to ensure the generalization ability of the model, while also making the model capable of accepting validation from independent external data. However, the existing studies mentioned above only used MVI as a separate task for prediction, ignoring the correlation between MVI and VETC. Based on the correlation and the complement supplied by VETC in predicting prognosis, coupled with the characteristics of the MTL model that could simultaneously predict related tags, the idea of MTL was adopted in our study. We integrated predicting MVI and VETC as two related tasks into a DL model at the same time. In the first stage of the feature extraction, we made the tasks share the same feature representation. In the second stage of the prediction, the feature representation was input into the targeted task prediction module, and the correlation between the tasks of predicting MVI and VETC could improve the prediction performance and generalization ability of the model. The difference between the two tasks and the respective predictive abilities of the two tasks in a targeted manner were ensured. Compared with the single-task model, the multitask model improved the performance of predicting MVI and the generalization ability of the model while outputting the results of VETC as well as the results of MVI, instead of separately training a model to predict VETC, thereby reducing the training time of the model. Finally, the MTL model improved the prediction accuracy of MVI from 0.85 in the original STL model to 0.9, and increased the AUC to 0.917 (95% CI: 0.825–1.000). By adding the task of VETC prediction, various other indicators, such as sensitivity, specificity, precision, and F1-score were improved, indicating that labels related to MVI indeed improved the prediction performance, and we considered that the potential connection between the labels was helpful.

As two important prognostic factors, MVI and VETC are both effective in prognostic stratification.^14^ Analogous results were obtained in our study. According to the difference in the MVI and VETC statuses in the results predicted by the 3D CNN, the patients were divided into four subpopulations. Survival analysis showed that stratification based on the prediction results was meaningful, indicating that the 3D CNN for MTL could evaluate prognosis by predicting the combination of MVI and VETC statuses. In particular, postoperative PFS and OS were significantly different between the VETC+/MVI+ group and other groups, such as the VETC−/MVI− and VETC+/MVI− groups, which indicated that our model has the potential to stratify patients with VETC+/MVI+ who have the shortest survival period. Although a significantly shorter median follow-up time was observed in patients with VETC+/MVI− and VETC−/MVI+ than in those with VETC−/MVI−, no significant differences in OS and PFS were detected between these groups, which might be caused by the small sample size. These results revealed that the coexistence of MVI and VETC could indeed shorten the OS and PFS, resulting in a poor prognosis. However, when one of the two existed, although the survival time was slightly prolonged, the stratification was still ambiguous. Collectively, these highlighted the potential prognostic value of the 3D CNN for MTL for personalized risk stratification and long-term management. However, the single institution, limited sample size, and strict exclusion standard limited the reproducibility and comparability of the study. Future multicenter studies with larger scale populations are needed to validate our findings. The data standardized module included in our model is prepared to accept data from other centers as an independent external validation panel to form multicenter studies. In the future, multi-center applications will be promoted with our 3D CNN framework as the core through the regionalized medical and telemedicine platforms we are building. In this way, a more convenient online system in the form of Browser/Server could be provided for other centers, the MRI images of patients could be uploaded using a Web browser for prediction, and the privacy of patient data would be protected by means of registration and encryption.

In conclusion, we proposed a DL model for MTL based on a 3D CNN and Gd-EOB-DTPA-enhanced MRI images for MVI and VETC prediction, which improved the prediction performance of MVI while assessing the VETC status of the sample. The outstanding performance of the model not only proved the correlation between MVI and VETC but also made the assessment of prognosis more accurate. An MVI/VETC remote prediction and diagnosis system based on our novel framework has potential to formulate treatment strategies and assess survival before surgery, and ultimately improve the survival time of HCC patients.

## Supplementary Information

Below is the link to the electronic supplementary material.

Supplementary file1 (PDF 121 kb)

## Data Availability

The data presented in this study are available on request from the corresponding author.
