# Supplementary material for: Application of a Convolutional Neural Network for Multitask Learning to Simultaneously Predict Microvascular Invasion and Vessels that Encapsulate Tumor Clusters in Hepatocellular Carcinoma
Source: Ann Surg Oncol. 2022 Jun 26;29(11):6774–83. doi: 10.1245/s10434-022-12000-6 (PMC9492610; doi:10.1245/s10434-022-12000-6)
Supplement: Supplementary file 1 — Supplementary file1 (PDF 121 kb) [file 10434_2022_12000_MOESM1_ESM.pdf]

Application of a Convolutional Neural Network for Multitask Learning to Simultaneously Predict Microvascular Invasion and Vessels that Encapsulate Tumor Clusters in Hepatocellular Carcinoma

Tongjia Chu, MD[1], Chen Zhao, MD [2], Jian Zhang, MD, PhD[1], Kehang Duan, MD [1], Mingyang Li, MD[3], Tianqi Zhang, MD[3], Shengnan Lv, MD[1], Huan Liu, MD, PhD[1], Feng Wei, MD, PhD[1*]

[1]Department of Hepatobiliary & Pancreatic Surgery, The First Hospital of Jilin University, Changchun, P.R. China;

[2]College of Computer Science and Technology, Jilin University, Changchun, P.R. China;

[3]Department of Radiology, The First Hospital of Jilin University, Changchun, P.R. China;

[*]Correspondence: wei_feng@jlu.edu.cn

Running Head

3D CNNs simultaneously predict MVI and VETC

Conflicts of Interest: The authors declare no conflict of interest.

Funding: This research is sponsored by grants from the National Natural Science Foundation of China (81972273), Jilin Provincial Medical Foundation (JLSWSRCZX2020-068), and Achievement Transformation Fund of the First Hospital of Jilin University (JDYYZH-2102035).

Supplementary Table 1. Parameters of three MRI scanners.

**Parameter**	**Discovery MR750 3.0 T**	**Ingenia 3.0 T**	**Achieva 3.0 T**
Echo time (TE) (ms)	1.67	1.4	1.43
Repetition time (TR) (ms)	4.06	3.3	3.03
Field of view (FOV) (mm)	360×290	400×304	375×298
Slice thickness (mm)	4.4	5	6
Slice gap (mm)	2.2	2.5	3
Flip angle (degrees)	10	10	10

Supplementary Table 2. Clinical characteristics of patients in training and validation cohorts. a. Microvascular invasion. b. Vessels that encapsulate tumor clusters.

a

Characteristics	Training		Pintra	Validation		Pintra	Pinter
	MVI-negative (58)	MVI-positive (35)		MVI-negative (24)	MVI-positive (16)		
**Age (y)***	56.14±10.349	54±9.762	0.393	58.33±8.442	56.56±7.545	0.502	0.371
**No. of male patients**	47(81.0)	31(88.6)	0.338	18(75.0)	15(93.8)	0.126	0.845
**No. of patients with hepatitis B virus infection**	43(74.1)	28(80.0)	0.519	20(83.3)	12(75.0)	0.519	0.644
**No. of patients with cirrhosis**	36(62.1)	21(60.0)	0.496	15(62.5)	9(56.3)	0.693	0.62
**Alanine aminotransaminase level (U/L)***	46.459±65.8186	49.603±48.8043	0.237	39.979±34.444	46.7±42.1645	0.74	0.833
**Aspartate aminotransaminase level (U/L)***	42.557±53.1813	45.171±36.5642	0.17	35.521±14.8335	56.438±50.3412	0.384	0.24
**γ-Glutamyltransferase (U/L)***	58.621±53.2417	90.7±92.2167	0.102	94.004±114.0559	118.331±124.6307	0.307	0.104
**a-Fetoprotein level (ng/ml)***	270.3878±472.22475	6480.2786±24874.19984	0.196	1715.5588±8179.638	1519.4375±4214.78432	0.09	0.105
**Alkaline phosphatase (U/L)***	79.231±23.4234	112.806±126.097	0.237	84.279±28.564	117.35±75.0942	0.112	0.232
**Serum total bilirubin (μmol/L)***	18.19±12.0034	17.026±7.4685	0.949	17.779±7.8638	19.7±7.8706	0.334	0.248
**Serum conjugated bilirubin (μmol/L) ***	5.559±7.2872	4.791±2.528	0.975	5.3±2.6891	6.106±2.7153	0.194	0.054
**Serum unconjugated bilirubin (μmol/L)***	12.631±6.8458	12.243±5.4231	0.975	12.479±5.4227	13.594±5.4602	0.529	0.418

b

Characteristics	Training		Pintra	Validation		Pintra	Pinter
	VETC-negative (62)	VETC-positive (31)		VETC-negative (27)	VETC-positive (13)		
**Age (y)***	56.29±10.73	53.42±8.663	0.289	58.44±8.64	55.92±6.626	0.488	0.371
**No. of male patients**	52(83.9)	26(83.9)	1	20(74.1)	13(100.0)	0.043	0.845
**No. of patients with hepatitis B virus infection**	46(74.2)	25(80.6)	0.49	22(81.5)	10(76.9)	0.736	0.644
**No. of patients with cirrhosis**	37(59.7)	20(64.5)	0.091	18(66.7)	6(46.2)	0.215	0.62
**Alanine aminotransaminase level (U/L)***	48.405±67.1146	46.116±42.1318	0.353	32.478±18.8306	63.831±55.1838	0.063	0.833
**Aspartate aminotransaminase level (U/L)***	45.763±55.9455	39.097±22.3407	0.328	33.219±12.6099	66.046±52.9808	0.035	0.24
**γ-Glutamyltransferase (U/L)***	57.008±52.0595	98.065±95.3964	0.008	92.774±109.0831	126.5±134.9434	0.254	0.104
**a-Fetoprotein level (ng/ml)***	2047.4026±13699.09686	3727.5252±18605.74808	0.067	168.66±627.94901	4686.9685±11605.63592	0.046	0.105
**Alkaline phosphatase (U/L)***	82.45±28.9172	110.7±132.7896	0.522	95.026±54.2832	102.662±55.3227	0.444	0.232
**Serum total bilirubin (µmol/L)***	18.316±11.458	16.623±8.3087	0.577	17.067±7.124	21.623±8.5888	0.097	0.248
**Serum conjugated bilirubin (µmol/L) ***	5.631±7.0923	4.548±2.3419	0.714	5.152±2.4251	6.6±3.0529	0.149	0.054
**Serum unconjugated bilirubin (µmol/L)***	12.685±6.4053	12.084±6.2299	0.56	11.915±5.0628	15.023±5.6588	0.119	0.418
